# Implementation of Artificial Intelligence in Writing Letters of Recommendation

**DOI:** 10.7759/cureus.94627

**Published:** 2025-10-15

**Authors:** Robert Snedegar, Courtney Pilkerton, Jun Xiang, Robert Allison

**Affiliations:** 1 Department of Family Medicine, West Virginia University School of Medicine, Morgantown, USA

**Keywords:** artificial intelligence, evaluation, letters of recommendation, medical education, residency

## Abstract

Introduction: Letters of recommendation (LoRs) play a critical role in the residency application process, yet their subjectivity raises concerns about reliability. Artificial intelligence (AI) offers a standardized alternative to human-generated LoRs. This study examines the quality of AI-generated LoRs compared to traditional LoRs.

Methods: Faculty at West Virginia University School of Medicine rated LoRs for residency candidates without knowledge that AI-generated letters were submitted. Candidate quality was controlled using pre-interview Thalamus (SJ MedConnect, Inc. dba ThalamusGME, Santa Clara, CA, US) scores. Independent sample t test and Kendall’s W test assessed differences in ratings and interrater agreement.

Results: AI-generated LoRs scored higher than traditional LoRs (4.14 vs. 3.29, p < 0.0001). For lower-quality candidates, AI LoRs significantly outperformed traditional LoRs (4.17 vs. 2.85, p < 0.0001). In higher-quality candidates, AI LoRs also scored higher (4.13 vs. 3.63, p = 0.006). Kendall’s W test demonstrated interrater concordance (p < 0.05).

Conclusions: AI-generated LoRs were rated superior to traditional LoRs, particularly for lower-quality candidates. These findings suggest AI could serve as an adjunct to enhance LoR quality and standardization. Further research is needed to explore ethical considerations and AI’s broader applicability in the letter-writing process.

## Introduction

Letters of recommendation (LoRs) are regarded as an important component of the residency application process. In a 2024 survey, residency program directors indicated that LoRs had an average importance rating of 4.2 on a five-point Likert scale in deciding which applicants to invite for an interview [1]. For medical students entering the National Resident Matching Program (NRMP), subjectivity in the interpretation of the writer’s narrative can impact the reviewer’s attitude toward candidates [2]. The number of comments referring to excellence in the Accreditation Council for Graduate Medical Education (ACGME) core competencies in LoRs may be useful for the stratification of applicants, but the objectivity of traditional LoRs can be influenced by several factors, particularly implicit racial biases [3-6].

The advent of artificial intelligence (AI) offers a promising alternative, with potential to standardize and streamline the LoR-writing process [7-9]. Multiple studies have suggested that AI tools can generate LoRs comparable to those written by humans, although skeptics still raise concerns about AI-generated hallucinations, inaccurate references, and inflated applicant characteristics [10-15]. For residency programs in family medicine, where interpersonal skills are paramount, understanding the nuances of AI versus traditional LoRs is particularly important.

The purpose of this study is to investigate the quality of AI-generated LoRs for fourth-year medical students entering the NRMP for family medicine as compared to their human-generated counterparts. We aim to address questions about the utility and implications of incorporating AI into the recommendation process.

## Materials and methods

Seven candidates selected for interviews with the West Virginia University Department of Family Medicine Residency from the 2023-24 NRMP Match Cycle were selected via a random number method. Ten faculty members from the department were contacted to complete a RedCap survey (described below), and all agreed to participate. No participants were informed that AI was used to generate letters prior to the study. All 10 survey participants had experience in interviewing candidates for the NRMP, ranging from two to 40 years of experience. Out of the 10 survey participants, eight completed the survey, and their responses were included in the data analysis.

For the seven selected candidates, two human-generated LoRs were pulled from their NRMP application and de-identified. For each of these candidates, investigators utilized OpenAI ChatGPT 3.5 (San Francisco, CA, US) with the prompt “Acting as a *** year physician, write me a letter of recommendation for a 4th year medical student applying for family medicine residency.” The de-identified qualifications of each applicant highlighted in their personal statement were then entered by the study authors into the prompt to create a LoR. All human and AI letters were de-identified. Each candidate had three (two human and one AI) LoRs to reflect the average number of LoRs submitted during the application process.

All AI- and human-generated LoRs were entered into a survey. Each LoR in the survey was accompanied by a single question asking “How would you rate the quality of this letter of recommendation?” accompanied by a five-point Likert scale (1: very poor; 2: poor; 3: adequate; 4: good; 5: excellent). Survey participants were sent emails asking them to participate in a project regarding what qualities make a good LoR, and all voiced willingness to participate. Reviewers received specific guidance to focus on the quality of the letter and not the quality of the candidate. The definition of “quality” was left to the discretion of the reviewer. Reviewers did not have any other application materials for the candidates.

The quality of each applicant was controlled for by using pre-interview Thalamus (SJ MedConnect, Inc. dba ThalamusGME, Santa Clara, CA) scores. Thalamus is a cloud-based software platform used by residency and fellowship programs to manage interview scheduling and assist in creating rank lists. Programs can tailor Thalamus scores to their preference in applicant qualities by weighing different categories differently. The candidates with a Thalamus score of ≥0.49 were grouped into the “higher quality” group, and the candidates with a Thalamus score of <0.49 were grouped into the “lower quality” group. The average Thalamus score of all candidates was 0.39; the range was 0.40.

All statistical tests in the current study were conducted using SAS (version 9.4, 2013, SAS Institute Inc., Cary, NC, US). We used the average scores of the two human-generated letters since there was a small but not significant difference between them. An independent sample t test was utilized to compare the average scores of the two human-generated LoRs and a LoR generated by AI for all applicants, as well as the stratified grouping of applicants (high quality and low quality) based on pre-interview Thalamus scores. Kendall’s W test was conducted to evaluate the agreement among eight faculty members for each human-generated LoR and the AI-generated LoR. All tests will be two-sided, and a p-value of less than 0.05 will be considered statistically significant. While this project involved human subjects, as an anonymous survey, this project was determined to be exempt from review by the West Virginia University Institutional Review Board (IRB) (protocol # 2402927280).

## Results

**Figure 1:**
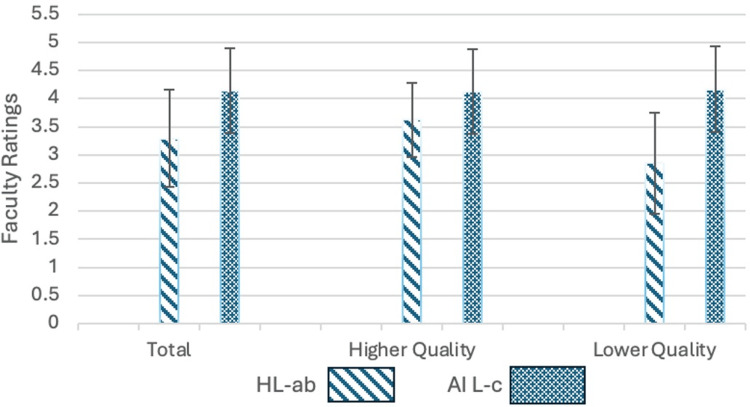
Mean Faculty-Rated Scores for Traditional and AI-Generated LoRs Scores based on a 5-point Likert scale (1: very poor; 2: poor; 3: adequate; 4: good; 5: excellent) LoRs: letters of recommendation

Results demonstrated statistically significant differences between mean scores of AI-generated LoRs as compared to human-generated LoRs, with AI earning a mean score of 4.14 compared to 3.29 for traditional LoRs (p < 0.0001). Concordance of scoring was observed among evaluators for each of the three letters (p < 0.05). Among students grouped in the “lower quality” group, AI scored an average of 4.17 as compared to 2.85 among traditional LoRs (p < 0.0001). For students in the “higher quality” group, AI scored 4.13 as compared to 3.63 among traditional LoRs (p = 0.006). A summary of the results is shown in Figure 1.

## Discussion

According to extensive PubMed and Google Scholar database searches, this is the first study to directly compare the perceived quality of AI-generated LoRs with their traditional, human-authored counterparts in the context of residency applications. Our findings indicate that AI-generated LoRs were rated more highly than traditional LoRs by experienced faculty reviewers, and this effect was consistent across both higher- and lower-quality applicants. Importantly, the margin of improvement was greatest among applicants rated in the “lower quality” category, suggesting that AI may amplify the strengths of applicants who might otherwise be disadvantaged by weaker human-authored letters (or that AI may inaccurately inflate the qualifications of these candidates). Additionally, the strong interrater reliability observed in our study reinforces the robustness of these findings, indicating that the preference for AI-generated letters was not driven by individual reviewer bias.

These results carry several important implications for medical education and the residency selection process. First, they highlight the potential of AI to bring greater standardization and equity to LoRs, a traditionally subjective component of residency applications that has long been criticized for variability, implicit bias, and inconsistent quality. By generating structured, comprehensive, and polished narratives, AI tools may help ensure that all applicants are represented fairly, particularly those from backgrounds or institutions where access to strong advocates may be limited. At the same time, however, the possibility that AI-generated LoRs could artificially inflate the apparent quality of weaker candidates raises questions about authenticity and the ability of LoRs to serve as meaningful differentiators between applicants. If AI tools are widely adopted without oversight, residency programs may need to reconsider the weight placed on LoRs in the selection process.

Our study also raises broader ethical and professional questions regarding authorship, transparency, and the role of faculty mentors in supporting students. LoRs are more than evaluative documents; they represent a professional endorsement and a reflection of the relationship between a faculty member and a trainee. If AI assumes a larger role in generating these letters, faculty members must remain actively engaged in reviewing, personalizing, and validating the content to ensure it reflects their true assessment of a candidate’s abilities. Otherwise, the value of LoRs may erode, and their function as a measure of mentorship and advocacy may be diminished. Currently, there is no formal guidance on the professional authorship of evaluative materials that comment on AI use from the NRMP or the Association of American Medical Colleges (AAMC). Future research should, therefore, explore not only the technical quality of AI-generated letters but also the ethical frameworks, disclosure practices, and best-practice guidelines that should accompany their use.

Our study is not without limitations. The small sample size, single-institution design, and program-specific values limit generalizability. Due to this, these results may be better defined as a pilot study. Furthermore, because reviewers were asked to evaluate “quality” without a standardized definition (since this is not explicitly defined at the institution where the study occurred), their judgments may not reflect how LoRs are interpreted among other selection committees. Another limitation is that the AI output is based on what the writer furnished to the AI as the basis of the LoR. Since only portions stressed in students’ applications were used as a prompt rather than the whole CV and personal statement, this could have resulted in unintentional variation of letter quality. Additionally, the question of what makes the AI-generated LoR of better quality (i.e., content, tone, etc.) remains unanswered. Despite these limitations, the strength of the findings suggests that AI warrants further exploration regarding its utility in professional authorship of evaluative materials, specifically its accuracy in representing candidates. Multi-institutional studies with larger samples, blinded evaluations across diverse specialties, and mixed-methods approaches simultaneously examining the accuracy of AI in accurately depicting candidates as compared to faculty viewpoints of the candidates' qualifications could provide deeper insights into both the advantages and pitfalls of AI-generated LoRs.

## Conclusions

This study, while limited by a small sample size, implies that AI has the potential to improve the quality and consistency of LoRs, offering a promising tool to address long-standing concerns about subjectivity and bias in the residency application process. However, its adoption must be accompanied by careful consideration of ethical, professional, and educational implications. Rather than replacing human-authored letters, AI may be best positioned as an adjunct that supports faculty in creating more equitable, higher-quality narratives. Future studies should continue exploring the quality of AI LoRs compared to traditional LoRs and the ethical implications of integrating AI into the interview process. Specifically, studies should focus on the accuracy of AI in depicting the quality of candidates in professional authorship of evaluative materials, whether the comfort level with using AI is advantageous in a residency candidate, and the ethical considerations of the continued integration of AI in the professional evaluation of medical students. As AI is increasingly integrated into society and residencies have access to more objective data through programs such as Thalamus, studies may also need to readdress the value of a LoR in the residency application process.
